# Clinical presentation of human African trypanosomiasis in Zambia is linked to the existence of strains of *Trypanosoma brucei rhodesiense* with varied virulence: two case reports

**DOI:** 10.1186/1752-1947-8-53

**Published:** 2014-02-14

**Authors:** Victor Mwanakasale, Peter Songolo, Olusegun Babaniyi, Pere Simarro

**Affiliations:** 1 Copperbelt University, School of Medicine, Ndola, Zambia; 2 World Health Organization, Country office, Lusaka, Zambia; 3 World Health Organization, Geneva, Switzerland

**Keywords:** Acute, Chronic, Control, HAT, Meningoencephalitic, Trypanosomiasis, Virulence

## Abstract

**Introduction:**

*Trypanosoma brucei rhodesiense* typically causes acute and severe human African trypanosomiasis in Zambia and other countries in Eastern and Southern Africa. Although a few atypical cases of chronic and mild forms of this disease were reported in Zambia more than 40 years ago, no such cases have been diagnosed over the last four decades.

**Case presentations:**

For the first case, a 19-year-old Black African woman from the Eastern Province of Zambia presented with symptoms and signs of an atypical chronic and mild form of the disease for a period of 2 years. For the second case, a 16-year-old Black African boy from the Northern Province presented with symptoms and signs of a typical acute and severe form of the disease for 3 weeks.

**Conclusion:**

Two strains of *T. b. rhodesiense* with varying degrees of virulence still do exist in Zambia. This has implications for control strategies at the national level.

## Introduction

Human African trypanosomiasis (HAT) is a re-emerging parasitic infection in Africa. In Zambia HAT is caused by a hemoflagellate of the species *Trypanosoma brucei rhodesiense*[1]. Transmission of this infection is by the bite of an infected tsetse fly of the genus *Glossina*[2]. This parasite typically causes an acute and severe form of HAT [3]. However, a few cases of a chronic and mild form of the disease have been reported in the past in Zambia [4]. The clinical presentation of the chronic and mild form of the disease resembles that of HAT caused by *Trypanosoma brucei gambiense* infection in West, Central, and parts of East Africa. In addition to Zambia, cases of chronic and mild *T. b. rhodesiense* infection have been reported in Malawi, which shares its western international boundary with Zambia [5].

Even though the number of cases of HAT has declined in recent decades, sporadic cases continue to be diagnosed from time to time in some regions of Zambia [6].

The aim of this article is to describe two different clinical presentations of HAT that imply the existence of two different strains of *T. b. rhodesiense*, one highly virulent and the other mildly virulent, in Zambia and to discuss the implications of this observation on HAT control strategies. To the best of our knowledge no cases of the chronic and mild form of this disease have been reported in Zambia in the past 40 years even though sporadic cases of acute and severe forms of the disease have been reported.

## Case presentations

### Case 1

A 19-year-old Black African woman from a settlement called Lumezi in the Lundazi district, Eastern Province of Zambia, presented at a local rural health center. Lumezi lies near the Zambia–Malawi border and close to Kasungu National Park in Malawi. Its coordinates are S12 30.436 E33 03.054. She complained of sleeping mainly during the day, heart palpitations, loss of weight, headache, body weakness, and feeling ‘blocked’ in the throat for 2 years. A thick blood film was prepared from her finger prick blood. The blood film was stained with Giemsa stain and examined for blood parasites on the same day. The blood film demonstrated the presence of trypanosomes but there were no malaria parasites. A lumbar puncture was also performed on the same day and her cerebral spinal fluid (CSF) examined microscopically; trypanosomes were found in her CSF.

She was then transferred to the local district hospital for admission and commencement of treatment. She was commenced on suramin as an in-patient at the hospital 28 days after first being seen at the rural health center. Initially she was given three doses of suramin at 1g/day intravenously on alternating days. From then on she received two doses of suramin at 1g/week. She was also commenced on melarsoprol at 120mg/day intravenously 3 days after her first dose of suramin. She received a total of 12 doses of melarsoprol. She responded well to treatment and showed no signs of side effects to the antitrypanosomal drugs. She was discharged from the hospital 14 days after admission. At the time of her discharge from the hospital she was said to be in a stable condition even though she complained of a slight headache. She was booked to be reviewed at a local rural health center for follow up assessment 3 months after discharge from the hospital.

### Case 2

A 16-year-old Black African boy was brought to a district hospital by his parents from Nabwalya area in the Mpika district, Northern Province of Zambia. The coordinates for Nabwalya are S12 25.117 E31 58.694; it is located in the Luangwa valley, between the North and South Luangwa National Parks of Zambia. He complained of fever, dizziness, sleeping mainly during the day, and constipation. These symptoms were of 1-week duration prior to his presentation at the hospital. He was examined by the medical doctor on duty 14 days later. A thick blood film was prepared using his finger prick blood. The blood film was stained with Giemsa stain and examined for blood parasites on the same day. The blood film demonstrated the presence of trypanosomes but no malaria parasites.

Because the hospital was not a treatment center for HAT he was referred to another hospital, which is the only treatment center for HAT in the district, 2 days later for admission and commencement of treatment. On admission at the hospital he had an altered level of consciousness. He could neither talk nor walk unaided. A lumbar puncture was requested and performed shortly after admission. A microscopic examination of his CSF revealed the presence of trypanosomes. He was commenced on suramin and melarsoprol intravenously 3 days later. A test dose of suramin at 0.25g was given on day 1. From then on he was given five doses of suramin at 1g/day, given as a single dose, on days 2, 4, 7, 12 and 19. He also received nine doses of melarsoprol at 126mg/day, given as a single dose, on days 1, 2, 3, 11, 12, 13, 21, 22, and 23. His general condition deteriorated on the 16th day of admission. He had not eaten during the previous three days. He did not talk, had swollen lips, and had tremors in his right hand which extended to his left hand. He died the following day, on the 17th day of admission.

## Discussion

The two case reports presented here demonstrate the existence of two strains of *T. b. rhodesiense* in Zambia. The first case was due to a mildly virulent strain of *T. b. rhodesiense* which caused the unusual chronic form of Rhodesian HAT whereas the second case was due to a highly virulent strain of *T. b. rhodesiense* and caused the usual acute and severe form of the disease. The first case was sick for 2 years prior to being diagnosed with the meningoencephalitic stage of HAT whereas the second case was sick for 3 weeks before being diagnosed with the meningoencephalitic stage of HAT.

Mild virulent strains of *T. b. rhodesiense* have been recently reported in Malawi [5]. The findings in the two case reports have updated previous observations on clinical features of HAT in Zambia [4] and stress the existence of two forms of Rhodesian HAT in the country. Therefore it would be worth conducting an active surveillance program for HAT in areas where mild and chronic cases of the disease have been reported in order to identify HAT carriers that would need early treatment. In areas where acute and severe forms of the disease have been reported, a passive surveillance program for HAT is appropriate to identify cases that would need treatment. In both situations frontline health workers need to have basic knowledge on the clinical presentation of HAT and the training for laboratory diagnosis of this disease [7], in addition to having functional laboratories and pharmacies well stocked with antitrypanosomal drugs.

## Conclusions

The two cases have demonstrated the existence of two strains of *T. b. rhodesiense* with varying virulence in Zambia. This has implications for the design of the national control program for HAT in Zambia.

## Consent

Written informed consent was obtained from the patient of the first case and from the father of the patient of the second case for publication of this case report. A copy of the written consents is available for review by the Editor-in-Chief of this journal.

## Abbreviations

CSF: Cerebral spinal fluid; HAT: Human African trypanosomiasis.

## Competing interests

The authors declare that they have no competing interests.

## Authors’ contributions

All the authors participated in the discussion of the two cases and contributed to the drafting of the manuscript. All authors read and approved the final manuscript.
